# What does my network learn? Assessing interpretability of deep learning for EEG

**DOI:** 10.1162/IMAG.a.1033

**Published:** 2025-12-02

**Authors:** Pinar Göktepe-Kavis, Florence M. Aellen, Sigurd L. Alnes, Athina Tzovara

**Affiliations:** Institute of Computer Science, University of Bern, Bern, Switzerland; Center for Experimental Neurology, Department of Neurology, Inselspital, Bern University Hospital and University of Bern, Bern, Switzerland

**Keywords:** EEG, decoding, deep learning, interpretability, feature visualization

## Abstract

Electrophysiological studies are profiting from multivariate pattern analysis methods. However, these mostly rely on machine-learning algorithms that assume consistent response latencies across trials and individuals. Deep learning provides high performance without such assumptions, but often at the cost of interpretability of learned features. Here, we evaluated how the interpretability of deep learning for electroencephalography (EEG) data is affected by preprocessing choices, the network’s architecture, and the way the learned features are extracted and visualized. We trained two convolutional neural networks (CNN): (1) ResNet, a residual network, and (2) EEGNet, which leverages spatiotemporal properties of EEG. We trained these networks to decode single-trial EEG responses to three different visual stimuli (visual dataset) and to the presence of a sound (auditory dataset). We then extracted and visualized learned features with two gradient-based techniques: saliency and gradient-weighted activation maps (GradCam). Results showed that EEGNet and ResNet performed at a similar level. Yet, visualization of learned features revealed that different architectures learn different aspects of the data. Between the two CNNs, EEGNet features had a higher similarity to the EEG data than ResNet features. Moreover, the latency and distribution of important electrodes varied depending on the visualization technique. GradCam provided features more similar to EEG data than those with saliency, emphasizing the impact of the feature extraction method on interpretability. Our results call for careful consideration of network architecture and feature visualization methods to improve interpretability, which is a crucial step for advancing the use of deep learning in EEG research.

## 1. Introduction

Deep learning algorithms have revolutionized the field of artificial intelligence, significantly advancing language predictions (Linzen & Baroni, 2021), or image processing and generation (Jiao & Zhao, 2019). The application of deep learning algorithms to time-domain signals like electroencephalography (EEG) remains, by comparison, limited. Fields of EEG research where deep learning algorithms have shown promising results include brain-computer interface (BCI) (Manor & Geva, 2015; Roy et al., 2019; Schirrmeister et al., 2017; Tibrewal et al., 2022) and automation of tedious tasks such as sleep scoring (Fiorillo et al., 2023). Deep learning has also been proposed as a promising venue for assisting in the diagnosis and prognosis of neurological disorders (Aellen et al., 2023; AlSharabi et al., 2023; Alves et al., 2022; Huggins et al., 2021; Jonas et al., 2019; Kim et al., 2023; Pham et al., 2022; Zubler & Tzovara, 2023). By comparison, the use of deep learning in fundamental neuroscience studies stays limited and is largely focused on the study of linguistic processing and predictions (Cai et al., 2025; Caucheteux et al., 2023; Goldstein et al., 2022, 2025; Riveland & Pouget, 2024; Russo et al., 2022; Tuckute et al., 2024).

Fundamental neuroscience studies rely in their vast majority on ‘traditional’ machine learning algorithms for performing multivariate pattern decoding, which identify the stimulus that resulted in a given neural response (Grootswagers et al., 2017). We speculate that one reason for this gap in the literature is the lack of understanding of how deep learning algorithms can be applied for decoding neural signals and their presumed lack of transparency. One factor driving the lack of transparency in deep learning applications is that of network architecture. One of the most commonly used architectures for EEG research are convolutional neural networks (CNNs) (Craik et al., 2019; Dixen et al., 2025; Ma et al., 2024; Rakhmatulin et al., 2024; Roy et al., 2019). Certain CNNs emphasize frequency decompositions (Sakhavi et al., 2015, 2018; Schirrmeister et al., 2017), or leverage the temporal dimension of EEG data (Köllőd et al., 2023; Lawhern et al., 2018), via temporal convolutions and filters, followed by spatial convolutions (Lawhern et al., 2018). CNNs that do not explicitly discriminate space from time, such as those developed for images (He et al., 2015; Krizhevsky et al., 2012; Simonyan & Zisserman, 2015; Szegedy et al., 2014), may perform well on EEG data (Aellen et al., 2021; Jonas et al., 2019; Zhao et al., 2023). However, they may be less sensitive to temporally evolving patterns of EEG activity. How the architecture of a CNN influences what parts of the EEG data are learned remains an open question.

Another aspect of interpretability, orthogonal to the choice of a network architecture, is our core ability to assess the features that a CNN has learned. Today, in the literature, there are several techniques to provide insights into what parts of the data CNNs are basing their decisions on. These span from gradient-based methods (Selvaraju et al., 2020; Simonyan et al., 2014; Springenberg et al., 2015; B. Zhou et al., 2016) to occlusion maps (Zeiler & Fergus, 2014), or correlations with a priori-defined features of the EEG signals (Schirrmeister et al., 2017). Gradient-based methods, relying on saliency maps, backpropagate a given data sample through a trained network and compute the gradient of a given class of interest with respect to the input data (Simonyan et al., 2014). Gradient-weighted Class Activation Map (GradCam) methods are based on similar principles but instead of backpropagating through the entire network, they only backpropagate up to a given convolutional layer (Selvaraju et al., 2020). In the case of images, saliency maps and GradCam have been shown to provide qualitatively different outputs (Selvaraju et al., 2020; Simonyan et al., 2014), which can further obscure the question of deep learning interpretability. Moreover, various layers of a convolutional network learn different levels of abstraction and thus also provide qualitatively different results (Selvaraju et al., 2020; Zeiler & Fergus, 2014). Although these discrepancies are well documented in imaging data, how the choice of a feature visualization method affects the extracted features for EEG-based decoding remains an open question. In one study using simulated EEG data, it was shown that the sensitivity and robustness of different methods vary across EEG features and noise levels (Sujatha Ravindran & Contreras-Vidal, 2023). In another, based on real EEG data, some methods were found to perform no better than random features when evaluated with perturbation and deletion tests (Cui et al., 2023). For emotion classification from EEG signals with CNNs, a recent study comparing five gradient-based approaches found different spatio-temporal patterns and feature importance, despite overlapping time periods linked to emotion processing in the brain (Mayor-Torres et al., 2023). These studies highlight the need for a thorough investigation of how the feature visualization method affects the interpretability in conjunction with other methodological choices and EEG characteristics.

Here, we aimed to systematically evaluate the interpretability of deep learning algorithms for EEG. We focused our investigations at two levels: (a) during training, on how the choice of a network architecture affects which information of the input data is learned; (b) after training, on how the way of extracting and visualizing learned features affects their interpretability. Our hypotheses were that different network architectures would result in accurate decoding, but the choice of network and feature extraction method would influence the network’s interpretability. We additionally hypothesized that architectures prioritizing the extraction of temporal information would result in more ‘interpretable’ features, likely reflecting known event-related potential (ERP) components.

## 2 Methodology

### 2.1 Datasets

We used two EEG datasets in two different sensory modalities: (1) a visual dataset and (2) an auditory dataset. Additionally, we validated the generalizability of the reported results in a third dataset, also in the visual modality (described in full detail in Haupt et al., 2025). Participants in all datasets reported normal or corrected-to-normal vision and normal hearing, and they had no history of psychiatric or neurological disorders. All participants signed a written consent form before the recordings, approved from the Ethics Committee of the Canton of Bern (2020-00060 and 2019-01651). Participant recruitment and further details about the control dataset are reported in Haupt et al. (2025).

#### 2.1.1 Visual dataset

This dataset consisted of EEG data recorded from 26 participants who were presented with three images (wood, flower, and apple) and reported in previous work (Göktepe-Kavis et al., 2024). Each image was presented for a duration of 0.3 s, followed by a white fixation cross of 1.5–2.5 s. Participants were instructed to fixate at the presented image and press a button when the fixation cross turned to red (10% of trials). Participants were presented with 300 image repetitions in total, 100 for each image.

#### 2.1.2 Auditory dataset

This dataset consisted of EEG recordings from 20 participants. The dataset included a pure tone condition, which was used in these analyses, as in previous work (Alnes et al., 2024). In the current study, we only included the EEG segments before and after the pure tones, as in previous work (Alnes et al., 2024) and focused on auditory responses in wakefulness.

#### 2.1.3 Control dataset

In addition to the two main datasets with typical datasets sizes (i.e., with ~20–25 participants), we used a larger dataset (with 43 participants and ~1024 trials per condition per participant, for a total of N = 88112 trials) that is publicly available (Haupt et al., 2025) as our control dataset. Participants were presented with animate (faces and animals) and inanimate images (places and objects) while their neural responses were recorded with EEG.

#### 2.1.4 Data preprocessing

Both EEG datasets were recorded with a 256-channel high-density Hydrogel Geodesic system (Luu & Ferree, 2005) at 1000 Hz sampling rate with Cz as an online reference. For consistency with previous work with these data, we kept the original preprocessing choices of each dataset (Alnes et al., 2024; Göktepe-Kavis et al., 2024). The visual dataset was downsampled to 256 Hz and filtered between 1–20 Hz. The auditory dataset was downsampled offline to 500 Hz and filtered between 1–40 Hz as in the previous report of this dataset (Alnes et al., 2024). Because some channels of the full 256 montage were recording primarily muscle activity, we used a reduced montage for the analysis, as in previous work, and retained 208 electrodes for the visual dataset (Göktepe-Kavis et al., 2024) and 173 for the auditory one (Alnes et al., 2024). ICA was applied to the remaining channels to exclude muscle artifacts, heartbeat, and ocular movements such as saccades and eye blinks. For the visual dataset, trials were extracted from 0 s up to 0.75 s relative to stimulus onset, and all trials after preprocessing per participant were retained, resulting in a total of 6066 trials in the dataset. For the auditory dataset, trials were extracted from the range of -0.5 s–0 s and 0 s–0.5 s relative to tone onset as pre-stimulus and post-stimulus trials, respectively. 400 trials per condition and participant (pre- and post-sound presentation) were randomly selected among ~2000 to reduce the computational cost of network training. The final dataset included a total of 16000 trials. Data were manually inspected for both datasets to exclude noisy trials. Data were re-referenced offline to the common average reference. All preprocessing was done using the MNE library (Gramfort et al., 2013).

### 2.2 Deep learning strategy

**Fig. 1.**
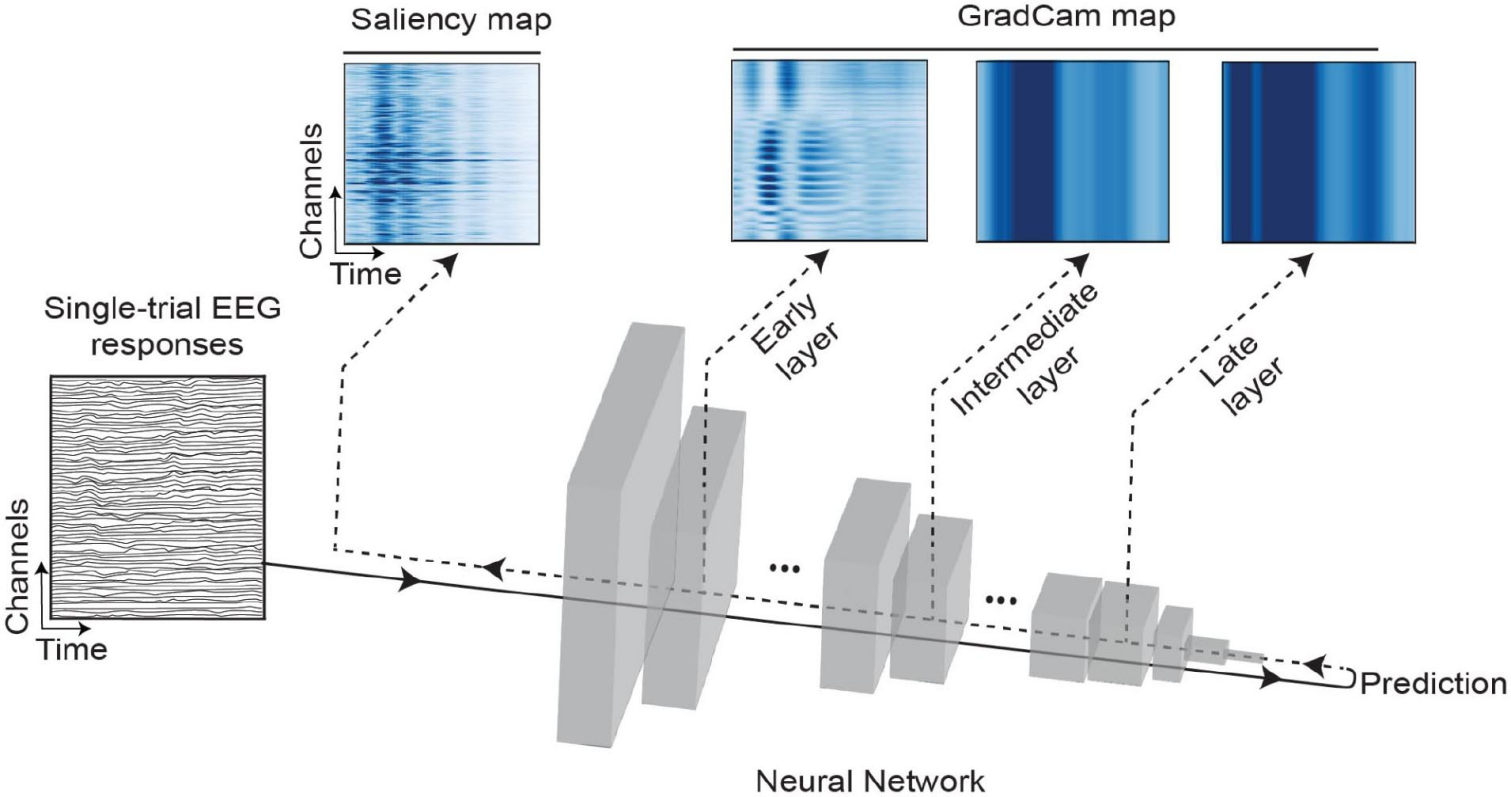
Analysis pipeline. Single-trial EEG responses were used to train CNNs to decode class-specific information. In this schematic, rectangular prisms represent convolutional layers of a CNN architecture (EEGNet and ResNet18 in the present study). After the input data passed through all the network layers, a class prediction was made. After training the CNNs, we extracted learned features using saliency maps (left heatmap above the neural network) and GradCam from an early, intermediate, and late layer of the CNNs (shown as heatmaps on the right above the neural network). Solid arrows passing through the network represent the feedforward flow of predictions, and dashed lines correspond to the backpropagation used to extract network features.

As a general approach, single-trial EEG responses of each dataset were used to train two different CNNs per dataset (Fig. 1 solid line). The training was done via 5-fold cross-validation. The participants were split into train-validation-test sets per fold, and their data were only used in the assigned set of a given fold, preventing trials from a participant appearing in multiple sets. Learning rate and optimizers were chosen for each network based on its performance on the training and validation sets of the visual dataset. Then, the same learning rates and optimizers were used for the auditory dataset to ensure the generalization of results. The final performance of each network was assessed at each fold by the respective test set, which consisted of participants who were not included in the training and validation sets of that fold. We ensured that results generalized to new and unseen data in two ways: (a) by evaluating network performance on test participants for the visual, auditory, and control datasets, which were not used to fine-tune any parameters; (b) by evaluating that the network architectures and optimization routine identified for the visual dataset generalized to the auditory one, and to the control dataset.

After training the networks, we inputted single-trial EEG responses from all participants to the trained networks to obtain predictions, which were then backpropagated through the network to extract learned features (Fig. 1 dashed lines). We extracted those in two ways: either at the network level (Fig. 1, top-left, saliency map), or at the early, intermediate, and late layers of the network (Fig. 1, top, GradCam maps). These analyses, including network training and feature extraction, were implemented in Python version 3.11.4, with TensorFlow version 2.13.0 (Abadi et al., 2015) and Cuda version 11.8 (NVIDIA and Vingelmann, et al., 2022).

We trained the networks with two tasks: for the visual dataset, we trained them to decode single-trial EEG responses to three presented images (wood, flower, and fruit). For the auditory dataset, we trained them to decode whether a given segment of EEG data was a pre- or post-stimulation segment. The reason for these two choices was to evaluate how the networks would perform in a ‘classical’ decoding scenario, where they are trained to decode EEG responses to different stimuli, and also in a more particular scenario where they decode response vs. no response, with the goal of evaluating whether the extracted features would also reflect that. In total, we included N = 6066 EEG single trials from N = 26 participants in the visual dataset and N = 16000 single trials from N = 20 participants in the auditory dataset.

#### 2.2.1 CNNs

We used two CNN architectures: (1) EEGNet (Lawhern et al., 2018) and (2) ResNet18 (He et al., 2015). These models were selected as they are among the most commonly used for EEG research and have shown robust training results in previous studies (Aellen et al., 2021; Jonas et al., 2019; Zhao et al., 2023). In order to compare the networks’ performance to ‘traditional’ decoding approaches, we additionally trained a baseline model of logistic regression, which is commonly used for decoding (Castegnetti et al., 2020; Hubbard et al., 2019; Kurth-Nelson et al., 2016; Liu et al., 2019)**.**

EEGNet is a CNN that was primarily designed for brain-computer interface applications (Lawhern et al., 2018). It leverages spatio-temporal properties of EEG signals by design: The network architecture consists of 3 convolutional layers, which can be considered relatively shallow compared to popular CNNs such as VGGNet (Simonyan & Zisserman, 2015) or ResNet (He et al., 2015), which have up to 18 or 152 convolutional layers. The first convolutional layer extracts features along the temporal dimension of EEG data, while the second one extracts spatial features along electrodes. In the last convolutional layer, spatio-temporal features are combined together. For our analyses, kernel sizes were set to 16 in the first convolutional layers and to 64 in the second convolutional layers, and dropout rate was set to 4, as in previous work (Aellen et al., 2023).

The second CNN, ResNet architecture (ResNet18), was originally designed for image processing (He et al., 2015), and also applied to EEG data (Aellen et al., 2021; Hasan et al., 2020). The version of ResNet we used here consists of 18 convolutional layers. The network parameters, such as kernel size of filters and max pooling kernels, were kept the same as in the original ResNet implementation (He et al., 2015), similar to previous work for EEG (Aellen et al., 2021).

#### 2.2.2 Baseline model

As a baseline model, we trained logistic regression in a time-point by time-point manner, as commonly done in EEG research (Castegnetti et al., 2020; Hogendoorn & Burkitt, 2018; Kurth-Nelson et al., 2016; Li et al., 2022; Liu et al., 2019). For comparison with CNNs, we report the maximum average decoding accuracy at any time point.

#### 2.2.3 Network training & evaluating performance

CNNs were trained by passing single-trial EEG data through the network, which, in turn, generated predictions about their class. The loss was quantified via categorical cross entropy:

$$L\;=\;-\sum_{c=1}^{C}t_{c}log(f{\left(y\right)}_{c})$$ (1)

where $t_{c}$ is one-hot encoding of the true label for class c, and $f{\left(y\right)}_{c}$ is the prediction of the network after applying a softmax function f. During training, the goal was to minimize the loss by updating the weights of the network via backpropagation. Decoding performance was measured via the categorical accuracy, which is the generalization of accuracy for one-hot encoded labels:

$$Accuracy\;=\frac{Correct\;predictions}{All\;predictions}$$ (2)

Learning rate and optimizers were selected separately for EEGNet and ResNet18 based on their performance only on the training and the validation sets of the visual dataset where our analyses were more exploratory. Based on initial analyses, the same learning rate and optimizers were used for the auditory dataset. As final performance in the main text we always report performance on the test sets.

We trained EEGNet with a stochastic gradient descent optimizer (Robbins & Monro, 1951) with a learning rate of 0.0001, with momentum set to 0.5, exponential decay of 0.94 in a staircase fashion, and batch size of 64. Stochastic gradient descent provided stable learning with good generalization, as previously reported (Keskar & Socher, 2017; P. Zhou et al., 2021). For ResNet18, we used the Adam optimizer (Kingma & Ba, 2017) as it is known to be strongly performant based on various classification tasks (J. Zhang et al., 2020). Here, we used Adam optimizer with a learning rate of 0.001 and a batch size of 64. We also implemented an early stopping method, such that training would stop if there was no decrease in validation loss in 25 consecutive training epochs, as in previous work (Aellen et al., 2021).

Both CNNs and logistic regression were trained in a 5-fold cross-validation. We split the participants in the datasets into training, validation, and test sets, which contained 81%, 10%, and 9% of all the available participants of a given dataset. We used this participant-based splitting approach to prevent data leakage and to ensure that models generalized to new participants. The train set was used to train the networks/model and the validation to fine-tune hyperparameters. The final decoding performance was assessed on the test set via categorical accuracy (formula (1)), which consisted of data from participants not included in the training and validation sets of the given fold. For consistency, we always used the same train/validation/test partitions of the data across both CNNs and logistic regression.

### 2.3 Feature extraction

Two gradient-based feature extraction algorithms were used to obtain insights into which parts of the EEG data were primarily relevant for the networks’ decoding: saliency maps (Simonyan et al., 2014) and GradCam (Selvaraju et al., 2020). Both algorithms are commonly used in the field of deep learning (Huff et al., 2021; Q. Zhang & Zhu, 2018) and have been previously used for EEG data (Aellen et al., 2021; Jonas et al., 2019) but have not been directly compared. Additionally, we employed a third method to extract features, Deep Learning Important FeaTures (DeepLIFT, Shrikumar et al., 2019, Supplementary Methods 1). As DeepLIFT is more recent, it has not been commonly used in EEG research. We thus test it on one dataset, where we show that it provides comparable features to saliency maps and GradCam (Supplementary Fig. 1).

For all of those methods, single-trial EEG data available from all participants in a dataset were inputted to a trained CNN to get the network’s predictions per trial. Trials that were correctly predicted were used for feature extraction. Extracted features were averaged first across trials, then across participants, and finally across cross-validation folds per experimental condition separately to obtain stable class-specific group-level features.

#### 2.3.1 Saliency maps

The algorithm for saliency map calculation computes the contribution of each dimension of the input data (i.e., channel by time-points) to the network’s output by backpropagating the network’s prediction to the data (Simonyan et al., 2014) following:

$$w\;=\;\frac{\varphi S_{c}\left(I\right)}{\varphi I}$$ (3)

where gradient *w* of the prediction score *S_c_*(*I*) was computed for class *c* with respect to the given input *I*. When an EEG signal is used as input, *w* represents the importance of the channel-time point pair for the network’s prediction. The resulting map gives insights into learned patterns at the level of the entire CNN.

#### 2.3.2 Gradient-weighted class activation maps (GradCam)

GradCam (Selvaraju et al., 2020) first computes the gradient of the network’s prediction score *S_c_(I)* for class *c* and input *I* with respect to feature map *A* of the convolutional layer of interest by backpropagating gradients to the layer of interest. Global average pooling is applied to the resulting gradients $w_{k}^{c}$ over the width and height of the feature map following:

$$W_{k}^{c}=\frac{1}{2}\sum_{i}\sum_{i}\frac{\varphi S_{c}(1)}{\varphi A_{k}}$$ (4)

where width, height, and the feature map at a convolutional layer were indexed with *i*, *j,* and *k*, respectively, and Z is the number of channel-time point pairs, resulting in an importance value per feature map (indexed with k). As the last step, each feature map is multiplied by the respective importance value, and all maps are combined linearly, followed by a rectified linear unit function (ReLU):

LGardCamc=ReLU(∑kwkcAk) (5)

The resulting map highlights the channel-time point pairs in the case of EEG data that positively contribute to the network’s output. This results in an importance weight per activation map. Then, the weighted activation maps are combined linearly, which provides a heatmap of learned features.

To assess how learned features visualized by GradCam changed across layers, we extracted features from an early layer (1^st^ convolutional layer), an intermediate layer (2^nd^ convolutional layer), and a late layer of the trained networks. As EEGNet consists of 3 convolutional layers, the visualization was done for each of them. As ResNet has 18 convolutional layers, for consistency, we selected three representative layers: 1^st^ and 2^nd^ as early and intermediate layers and the 10^th^ convolutional layer as the late layer, as layers after that resulted in zero gradients, possibly due to the deep architecture of ResNet.

### 2.4 Feature visualization

After extracting the learned features by the two networks with saliency and GradCam algorithms, we visualized them in three complementary ways: (a) as time by electrode heatmaps, which is the closest to the output of these techniques; (b) as time-courses of virtual EEG electrodes; and (c) as topographic maps, which are close to how EEG data are conventionally visualized. To obtain the EEG-like feature visualization, we transformed the ‘raw’ features with a surface Laplacian (regularization parameter *lambda2* = 10^-5^, *stiffness* = 4.7), as is common in the field of EEG research to smooth topographic representations (Kayser & Tenke, 2015).

### 2.5 Network interpretability score

To quantify the interpretability of CNNs, we calculated the similarity between EEG data and the features learned by CNNs, via Pearson’s correlation of each participant’s data and features. As the feature maps without the laplacian transform have only positive values, we correlated those with the absolute amplitude of EEG responses. We computed this correlation over time for each participant. To quantify interpretability at the group level, the resulting time courses of correlation scores were statistically compared against zero, which indicates no correlation via nonparametric Wilcoxon signed-rank tests at each time point. We used false discovery rate (FDR) to correct for multiple comparisons. We also extracted the maximum interpretability score and its latency for each participant per network and feature extraction method. For each network, we pairwise compared the maximum scores achieved by different methods and layers as well as their latencies using non-parametric Wilcoxon signed-rank tests.

## 3 Results

### 3.1 Decoding results

#### 3.1.1 Visual dataset

**Fig. 2.**
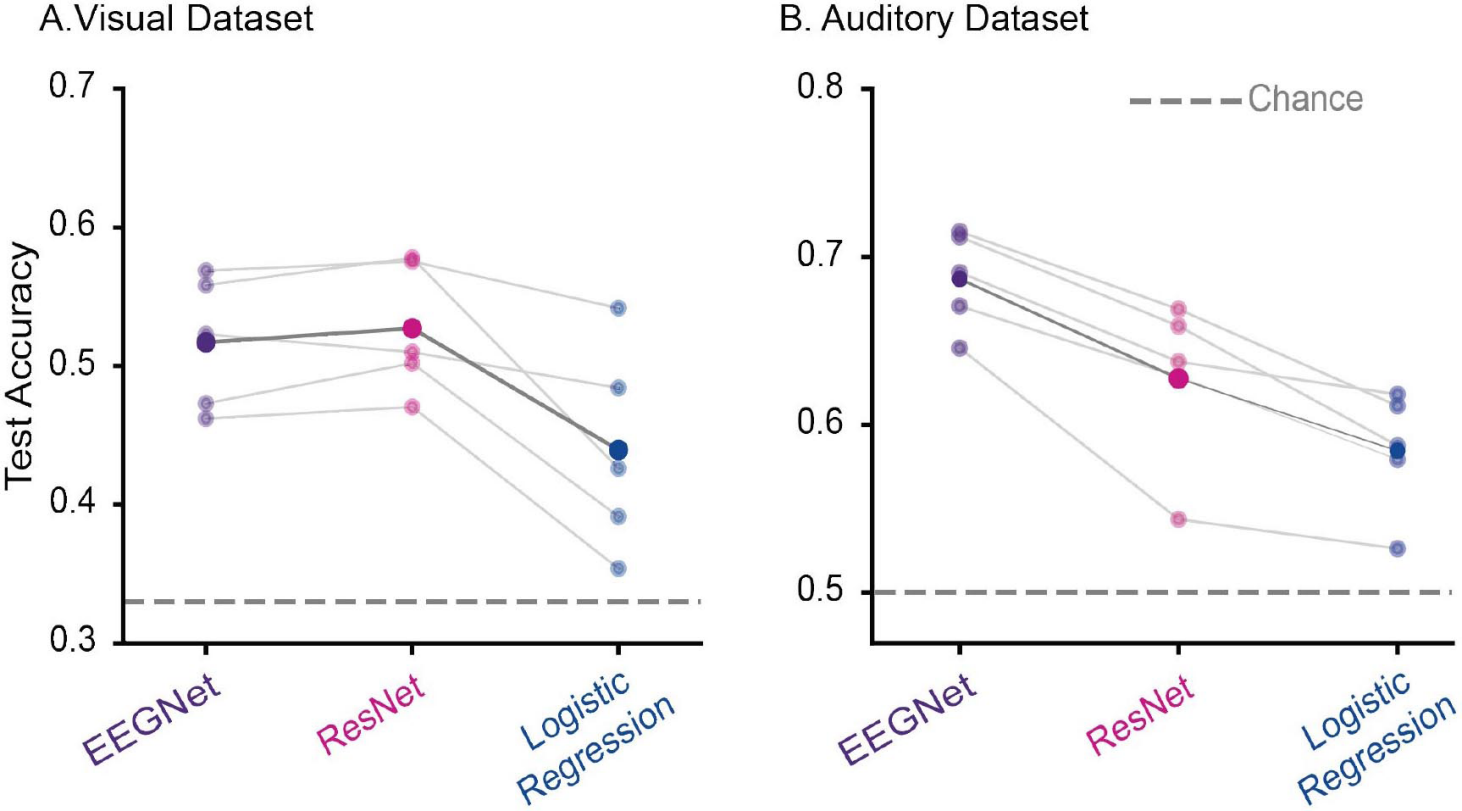
Decoding results of EEGNet, ResNet, and logistic regression models. (A) 3-class decoding for the visual dataset and (B) 2-class decoding for the auditory dataset. Each small (semi-transparent) dot represents the accuracy score obtained for test set data in one cross-validation fold, the big opaque dot represents the mean of all folds, and the gray dash line indicates chance level (0.33 for the visual dataset and 0.5 for the auditory dataset). Purple, pink, and blue colors were used for EEGNet, ResNet, and logistic regression models, respectively.

The average 3-class decoding performance for EEGNet on the test set across all folds was 0.52 ± 0.04 (Fig. 2A, Supplementary Table 1 reporting also train and validation sets for completeness). ResNet18 reached an average decoding performance similar to EEGNet of 0.53 ± 0.04. The logistic regression algorithm achieved a maximum decoding performance of 0.44 ± 0.07. The performance of both CNNs and logistic regression was higher than the empirical chance level, which in the case of 3-class decoding corresponds to 0.33 (Fig. 2A), showing that stimulus identity can be decoded from single-trial EEG responses.

#### 3.1.2 Auditory dataset

For the auditory dataset, the 2-class decoding performance of EEGNet was 0.69 ± 0.03 across folds, while ResNet performed at 0.63 ± 0.04 (Fig. 2B, Supplementary Table 1). The maximum accuracy achieved by logistic regression was 0.59 ± 0.03. The empirical chance level was 0.5, suggesting that all decoding approaches provided above-chance level performance. A statistical comparison of the decoding results across CNNs and logistic regression was not possible due to the limited number of validation folds. Yet, EEGNet slightly exceeded the decoding performance achieved by ResNet, while both networks majorly outperformed logistic regression (Fig. 2B).

#### 3.1.3 Control analysis

To ensure that the presented results generalize to data acquired in different experimental settings, we further tested the generalizability of the training pipeline in an additional, control dataset. This included a 2-class decoding task of discriminating EEG responses to animate vs. inanimate images (Haupt et al., 2025, control dataset, Supplementary Fig. 2). EEGNet achieved a mean accuracy of 0.67 ± 0.09, ResNet 0.64 ± 0.018, and logistic regressions 0.61 ± 0.003 while the chance level was 0.5 (Supplementary Fig. 2), successfully differentiating between the animate and inanimate image conditions from EEG responses. Consistent with results from the visual and auditory datasets, the CNN models performed better than the logistic regression, demonstrating their effectiveness in decoding neural signals across datasets.

### 3.2 Learned features

#### 3.2.1 Features for the visual dataset

**Fig. 3.**
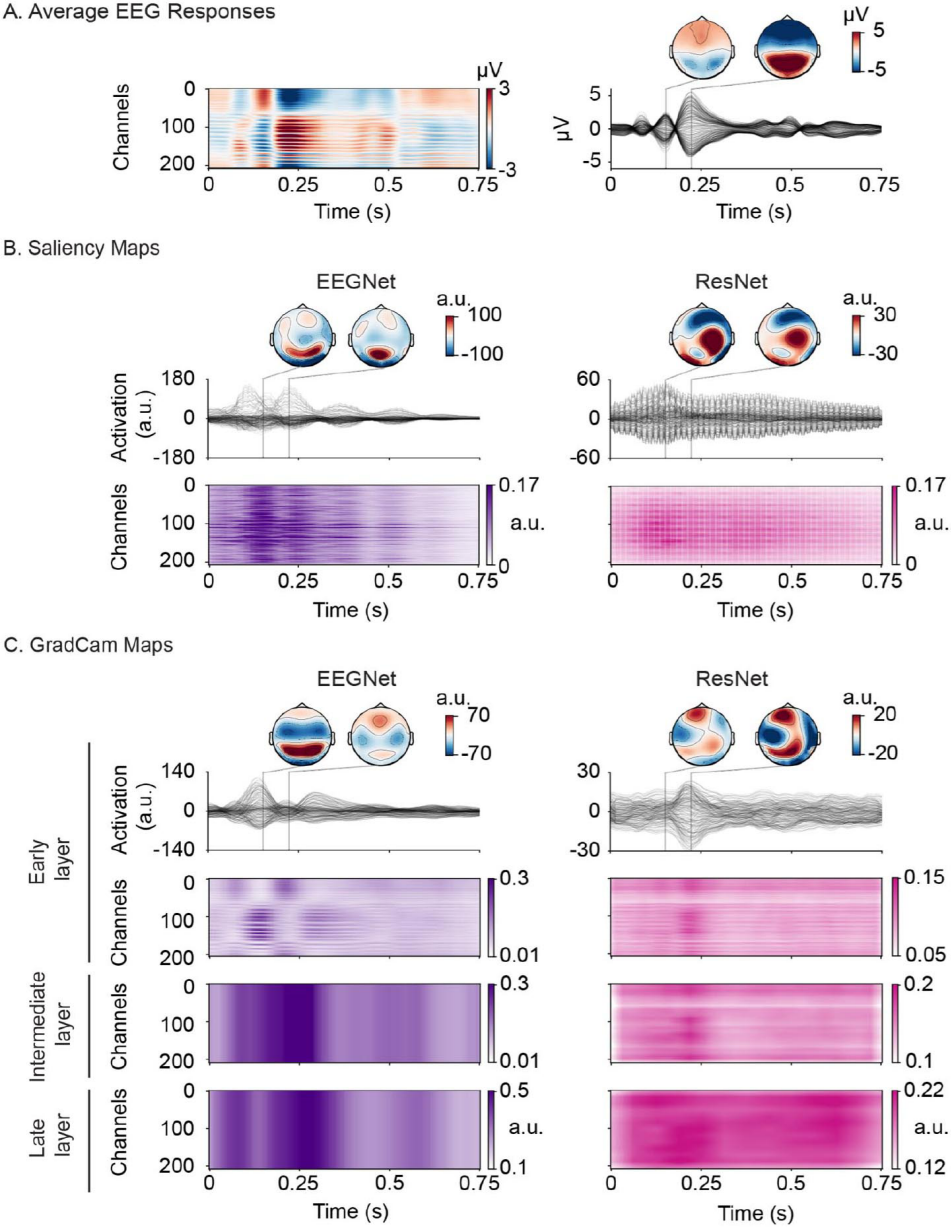
Feature visualization for the visual dataset. (A) Grand average EEG responses to the visual stimuli, shown as a heatmap and butterfly plot with corresponding topographic maps. (B-C) Visualization of learned features extracted with (B) saliency and (C) GradCam for EEGNet (left, purple) and ResNet (right, pink). GradCam extracted features from early, intermediate, and late layers. Intermediate and late layers were visualized as heatmaps (C, last two rows). Darker colors corresponding to higher saliency values indicate important channel-time point pairs for the network’s prediction on the visual dataset. The values plotted as topographic maps have been transformed with surface Laplacian to emphasize spatial patterns.

We extracted the features learned by EEGNet and ResNet using two algorithms: saliency maps (Simonyan et al., 2014) and GradCam (Selvaraju et al., 2020). On the visual dataset (Fig. 3), saliency maps and early layer GradCam revealed that both CNNs particularly focused on the first 0.3 s of the EEG response (Fig. 3B-C, heatmaps and butterfly plots; Supplementary Fig. 3), which corresponds to the time period in which EEG responses to the visual stimuli peaked across channels (Fig. 3A, 0.15 s and 0.22 s). This also corresponds to known ERP components of visual processing, including the P1 and N1 (Hopf et al., 2002; Ritter et al., 1983) as well as the typical latency range for neural decoding of objects (Contini et al., 2017), suggesting that both networks learned electrophysiologically relevant patterns.

Yet, the topographic representation of learned features (shown at the peak of EEG responses at 0.15 s and 0.22 s, Fig. 3A, right) revealed different spatial patterns depending on the network architecture (Fig. 3B-C), which changed over time (Fig. 3B-C, topographic maps). Both with saliency (Fig. 3B, left) and GradCam (Fig. 3C, left, early layer), EEGNet features had their highest values on occipital EEG channels (replicated by an alternative technique, DeepLift, as a control analysis, Supplementary Fig. 1), which typically show strong responses to visual stimuli (Hopf et al., 2002; Proverbio et al., 2007; Rousselet et al., 2007). ResNet features combined occipital and frontal channels when estimated with GradCam (Fig. 3C, right) and central channels when estimated with saliency maps (Fig. 3B, right).

#### 3.2.2 Features for the auditory dataset

**Fig. 4.**
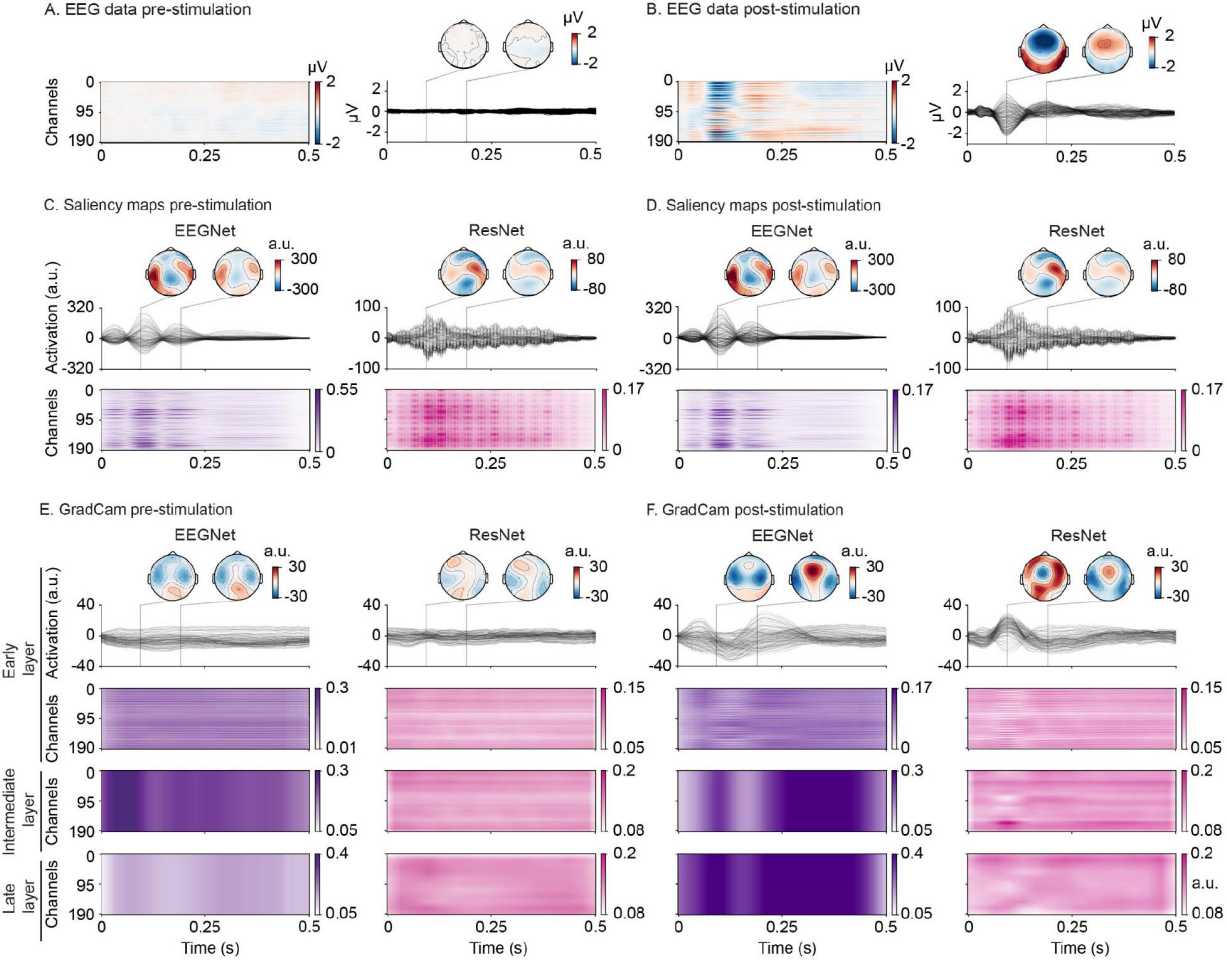
Feature visualization for the auditory dataset. (A-B) Grand average EEG data for (A) pre- and (B) post-stimulation intervals. (C-D) Visualization of EEGNet (left, purple) and ResNet (right, pink) features extracted with saliency maps for (C) pre-stimulation and (D) post-stimulation intervals. (E-F) Visualization of EEGNet (left, purple) and ResNet (right, pink) features extracted with GradCam for (E) pre-stimulation and (F) post-stimulation intervals. GradCam extracted features are displayed for both architectures in early (first row), intermediate, and late (third row) layers. Intermediate and late layers were visualized only as heatmaps (last two rows). Darker colors indicate higher saliency or GradCam values, corresponding to higher relevance in channel-time point pairs for the network’s prediction on the auditory dataset. The values plotted as topographic maps have been transformed with surface Laplacian to emphasize spatial patterns.

On the auditory dataset, where the networks were trained to decode pre- vs. post-stimulation intervals (Fig. 4A-B), we visualized the learned features by experimental condition separately to assess how the networks represent the presence and absence of auditory stimulation. Features extracted with saliency maps for the pre-stimulation condition retained spatiotemporal patterns that resembled qualitatively the ones in response to the sounds but with slightly lower intensity (Fig. 4C). Conversely, GradCam revealed diffuse features with low values across time and channels and little fluctuations for both networks, similar to what was observed in the average EEG responses (Fig. 4A).

For the post-stimulation condition, both networks showed the strongest features within the first 0.2 s after sound onset (Fig. 4D and F, early layer, Supplementary Fig. 6, right). This latency matched the peak of EEG responses (Fig. 4B, right, at 0.094 s) and corresponds to the N100, a common component of auditory processing (Hari et al., 1980; Näätänen & Picton, 1987; Zouridakis et al., 1998). Similar to the visual dataset, spatial patterns of the learned features differed between EEGNet and ResNet. EEGNet features included temporal (with saliency, Fig. 4D) and frontal (with GradCam, Fig. 4F, early layer) EEG channels. ResNet’s features extracted as saliency maps highlighted temporo-parietal channels (Fig. 4D, right). When extracted via GradCam, they highlighted more diffuse patterns, including frontal, temporal, and occipital EEG channels (Fig. 4F, right). Extracted features from deeper layers revealed that temporal and spatial resolution was lost, while resemblance to the EEG data was minimal (ResNet) or none (EEGNet) (Fig. 4F, last two rows).

### 3.3 Network interpretability

**Fig. 5.**
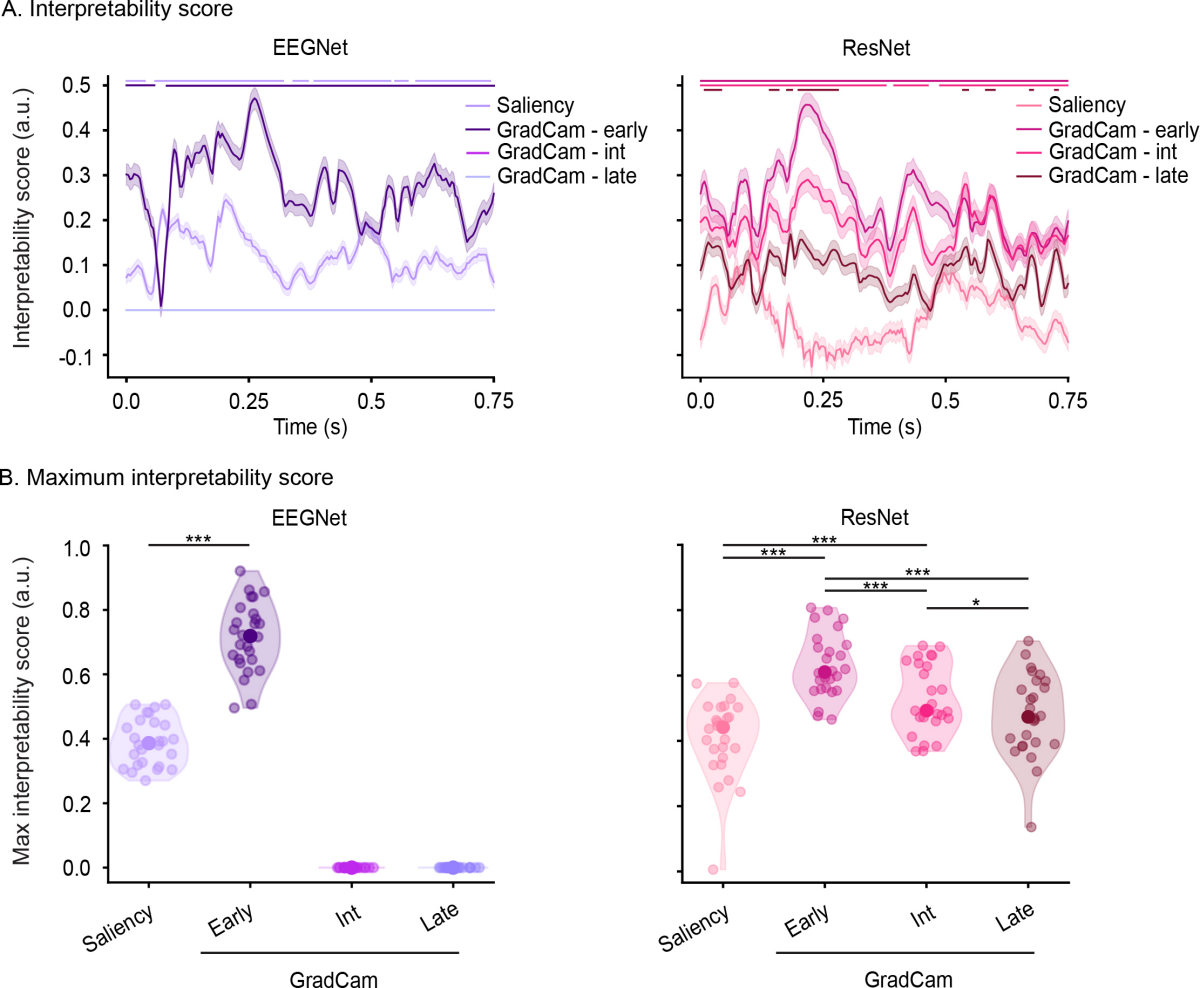
Interpretability score for the visual dataset. (A) Time course of interpretability scores of features extracted by saliency and GradCam from early, intermediate (int), and late layers. Horizontal bars indicate the time points significantly different from zero after FDR correction (p < 0.05, non-parametric Wilcoxon signed-rank test). (B) Maximum interpretability scores per network and feature visualization. Each dot in panel (B) corresponds to one participant. Scores of each feature extraction method and layer were statistically compared using the Wilcoxon paired-rank test (*p < 0.05, ***p < 0.001). In all panels, purple and pink colors represent EEGNet (left) and ResNet (right), respectively.

For both the visual and auditory datasets, the maximum interpretability scores (i.e., similarity between features and EEG data) were obtained for EEGNet and early layers of GradCam (Fig. 5, Supplementary Figs. 4 and 5 comparing it to DeepLift as a control analysis). A high interpretability score, significantly different from zero, persisted for almost the entire time course of the post-stimulus interval for the visual dataset (Fig. 5A, left, p_corr_ < 0.05) and for two distinct clusters for the auditory dataset (Supplementary Fig. 4A, left, p_corr_ < 0.05, the first 0.11 s and from 0.18 to 0.44 s post-sound onset).

**Fig. 6.**
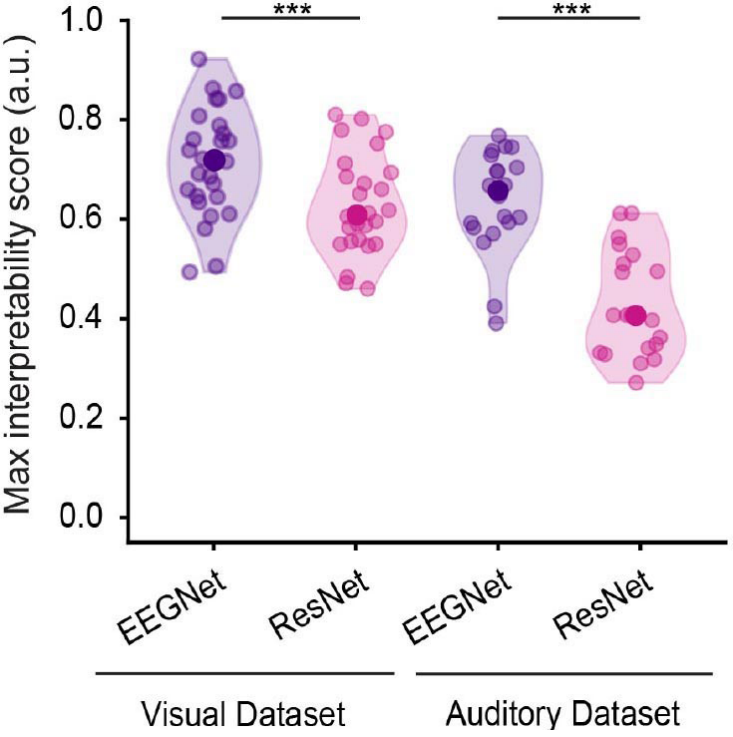
Overview of maximum interpretability scores across networks and datasets of early layer features extracted with GradCam for visual (left) and auditory (right) datasets. Each dot corresponds to one participant. EEGNet and ResNet scores were statistically compared using the Wilcoxon paired-rank test (***p < 0.001). Purple and pink colors represent EEGNet and ResNet, respectively.

For both datasets, the second highest interpretability scores after early layers of GradCam were obtained with saliency maps (Fig. 5 and Supplementary Fig. 4, left), while late layers of GradCam provided very low scores for EEGNet (Fig. 5 and Supplementary Fig. 4, left) and not that different scores for ResNet compared to early ones (Fig. 5 and Supplementary Fig. 4, right, Table 1 for full report of scores, Table 2 for statistical comparisons, Supplementary Table 2 and Supplementary Fig. 6 for latencies). Moreover, significantly higher interpretability scores were achieved by EEGNet compared to ResNet for both datasets (Fig. 6, z = -3.81, z = -4.08, p < 0.001 for the visual dataset (left) and the auditory (right)).

**Table 1.** Maximum interpretability scores across CNNs and feature extraction methods and layers for the (top row) visual and (bottom row) auditory datasets.

	EEGNet				ResNet			
	Saliency	GradCam Early	GradCam Int	GradCam Late	Saliency	GradCam Early	GradCam Int	GradCam Late
Visual dataset	0.39 ± 0.01	0.72 ± 0.02	4 x 10^-16^ ± 0.00	4.2 x 10^-16^ ± 0.00	0.41 ± 0.02	0.63 ± 0.02	0.53 ± 0.02	0.48 ± 0.03
Auditory dataset	0.32 ± 0.02	0.64 ± 0.02	4.4 x 10^-16^ ± 0.00	4.3 x 10^-17^ ± 0.00	0.39 ± 0.02	0.43 ± 0.02	0.49 ± 0.02	0.48 ± 0.02

Reported values are mean ± standard error across participants.

**Table 2.** Statistical comparisons of maximum interpretability scores across feature extraction methods and layers for each CNN for the (left) visual and (right) auditory datasets.

	Visual dataset		Auditory dataset	
	EEGNet	ResNet	EEGNet	ResNet
Saliency vs GradCam-Early	z = -4.46 p < 0.00001	z = -4.36 p < 0.0001	z = -3.9 p < 0.0001	z = -1.20 p > 0.05
Saliency vs GradCam-Int	z = -4.46 p < 0.00001	z = -2.88 p < 0.005	z = -3.9 p < 0.0001	z = -2.43 p < 0.05
Saliency vs GradCam-Late	z = -4.46 p < 0.00001	z = -1.69 p > 0.05	z = -3.9 p < 0.0001	z = -2.54 p < 0.05
GradCam Early vs Int	z = -4.46 p < 0.00001	z = -4.13 p < 0.0001	z = -3.9 p < 0.0001	z = -2.95 p < 0.005
GradCam Early vs Late	z = -4.46 p < 0.00001	z = -4.25 p < 0.0001	z = -3.9 p < 0.0001	z = -1.38 p > 0.05
GradCamInt vs Late	z = -1.20 p > 0.05	z = -2.43 p < 0.05	z = -0.47 p > 0.05	z = -0.11 p > 0.05

## 4 Discussion

In this study, we investigated how the interpretability of deep learning models for EEG decoding is affected by methodological parameters including network architecture and feature extraction method. We trained two well-known CNNs with different architectures, EEGNet and ResNet, to decode the identity of visual stimuli or the presence or absence of auditory stimulation. To assess interpretability, we extracted learned features from the trained CNNs using saliency maps and GradCam. Despite largely similar decoding performance for both CNNs, they learned different spatial distributions of the EEG data, albeit at similar latencies. GradCam computed in early layers consistently provided higher interpretability scores than saliency maps, regardless of CNN architecture or dataset. Overall, our findings support the strong potential of CNNs in fundamental EEG research, while being mindful of the chosen network architecture and feature visualization techniques, in order to improve interpretability.

### 4.1 Deep learning for EEG decoding

Decoding has become a prominent tool for analyzing neural patterns across various research areas in neuroscience, spanning from sensory perception (Contini et al., 2017; Isik et al., 2014; King et al., 2014; Tzovara et al., 2012) to decision-making (Castegnetti et al., 2020; Tzovara et al., 2012, 2015; Wimmer et al., 2023) and memory consolidation (Kurth-Nelson et al., 2016; Liu et al., 2019). These studies typically employ traditional decoding methods in a time-locked manner, assuming time- and space-invariant neural responses, and are thus susceptible to inter-individual and inter-trial variability in response characteristics. Deep learning offers a time- and space-invariant solution that has shown great potential to improve the decoding performance for EEG (Aellen et al., 2021; Tabar & Halici, 2016; Tang et al., 2017; Tibrewal et al., 2022). However, the feasibility of deep learning algorithms for EEG decoding is often a concern due to relatively small sample sizes compared to computer vision or language applications. Therefore, further studies are necessary to validate deep learning models and assess their applicability in experimental designs and participant cohorts typical of EEG research. Here, we focused on EEGNet and ResNet which are relatively established for brain computer interface, or clinical research purposes, but are not used in fundamental neuroscience studies for EEG-based decoding. We first assessed the performance of the CNNs on two typical neuroscience datasets (i.e., with ~20 participants, with 6’066 and 16’000 single-trial EEG responses in total) and also on an open-access, control dataset (with 43 participants, 88’112 single-trial responses in total) as an additional control analysis. These datasets differed in modality, task, recorded participants, and recording laboratory, and were selected to develop our analysis pipeline (visual dataset), and to validate the robustness of the employed models in a dataset recorded in the same laboratory but different modality (auditory dataset) and also in a dataset recorded by a different laboratory and openly released (control dataset, Haupt et al., 2025). These datasets have sample sizes typical for current EEG research, ensuring that our findings are applicable to standard experimental setups. In all three datasets, we consistently showed that both tested CNNs outperformed a traditional decoding analysis based on logistic regression. These results suggest that CNNs can effectively decode single-trial EEG responses collected for cognitive neuroscience tasks, in accordance with previous work (Aellen et al., 2021; Farahat et al., 2019; Naveilhan et al., 2025; F. Wang et al., 2018).

### 4.2 What do deep learning algorithms learn from EEG data?

One main criticism for a broader use of deep learning in EEG research arises from their black-box approach. This follows not only from the complex network architecture but also from the method used to visualize learned patterns in the data.

Here, we found that the most important features for decoding were primarily present in the first 0.3 s and 0.2 s after the stimulus onset for the visual and auditory datasets, respectively. These latencies were consistent across feature extraction methods and network architectures and are electrophysiologically relevant. For the visual dataset, the most relevant period for decoding was the first 0.3 s post-stimulus onset which includes the P1 and the P2 components, which are associated with basic stimulus processing (Ritter et al., 1983; C. Wang et al., 2012) and higher-order category-specific processing (Kiefer, 2001; Proverbio et al., 2007; Rousselet et al., 2007). In auditory processing, the first 0.2 s after stimulus onset encompass the P1 or N1, which are associated with early auditory processing (Hari et al., 1980; Näätänen & Picton, 1987; Zouridakis et al., 1998). These findings indicate that both CNN architectures and feature extraction methods provide temporally and electrophysiologically relevant features.

The main difference in the features learned by the two networks laid in their spatial patterns. In the visual domain, EEGNet’s strongest features with saliency maps and GradCam were mainly observed in the occipital channels, aligning with existing literature on visual processing and category discrimination (Kiefer, 2001; C. Wang et al., 2012). On the other hand, ResNet’s features were found in parietal and frontal channels, which do not correspond to the ones typically expected given the decoding task and sensory modality. For the auditory dataset, EEGNet’s topographic representation revealed temporal and frontal electrodes using saliency and GradCam, respectively. These align with existing literature on auditory processing (Fogarty et al., 2020; Hari et al., 1980; Näätänen & Picton, 1987). By contrast, ResNet’s features were a mixture of temporal, frontal, and occipital electrodes, which cannot be directly linked to existing literature. These results suggest that architectures utilizing spatiotemporal properties of EEG signals, like EEGNet, may provide more neuroscientifically relevant features.

Regardless of network architecture, features visualized with GradCam from early CNN layers had the closest resemblance to the EEG data (also confirmed by the control analysis comparing GardCam with DeepLift, Supplementary Fig. 5). This is partially expected, as this layer is the closest to the input data. Nevertheless, previous studies that used GradCam mostly opted for the last convolutional layer as these features could potentially be more representative of the network’s abstraction and final decision (Jonas et al., 2019; Selvaraju et al., 2020). Here, we confirm this result by showing that late layer features visualized by GradCam were, indeed, very abstract (Figs. 3C and 4F) and contain little to no spatial granularity, but mainly temporal information. This is consistent with the fact that the last CNN layers provide the most abstract features, resembling the visual processing hierarchy (Mahendran & Vedaldi, 2015; Yosinski et al., 2015). Our results suggest that early layers could be suitable candidates for extracting features that offer a neuroscientifically relevant interpretation, while late layers of a network can provide higher levels of abstraction. The interpretability of different layers, as shown through our comparisons between EEGNet and ResNet, is closely linked to network architecture. Changes in the layer organization of existing architectures, for example, the order of temporal versus spatial convolutions in EEGNet, can influence interpretability. As most EEG studies rely on existing architectures without modification (Aellen et al., 2021; Mahjoory et al., 2024; Naveilhan et al., 2025), we focused our analysis on the original network designs. Future work could extend this by systematically reorganizing model architectures to assess how such structural changes impact interpretability.

The task of decoding pre- vs. post-stimulation periods in EEG data raises questions about what CNNs learn in the absence of a stimulus. Despite the absence of auditory stimulation, saliency maps for pre-stimulation intervals showed a spatial distribution and latencies similar to post-stimulation ones. In contrast, GradCam extracted diffused features for pre-stimulation, which were distinct from those for post-stimulation. This discrepancy likely arises from algorithmic differences: GradCam focuses on channel-time point pairs that only positively contribute to class predictions (Selvaraju et al., 2020), avoiding gradient leakage across conditions, whereas saliency maps highlight where in the input data the network focuses on (Simonyan et al., 2014).

In this study, we defined interpretability as a resemblance between extracted features and EEG data. We opted for a similarity-based approach to validate whether deep learning algorithms effectively learn electrophysiologically relevant patterns from the input EEG data. However, deep learning may also extract other features that are currently unknown in existing EEG literature (Vahid et al., 2020). Thus, a low interpretability score does not necessarily imply that the network learned irrelevant information but rather that the information was not similar to average EEG responses. This may be due to the extraction of non-linearities or amplification of patterns that are present in single-trial EEG responses but lost when averaging. We expect that as the use of deep learning for EEG research gains momentum, both known and novel electrophysiological components may arise. This, in turn, can help identify new patterns in EEG datasets, thereby improving our understanding of neurocognitive mechanisms in the healthy and pathological brain.

## 5 Conclusion

In summary, we studied how, related to network architecture and feature extraction affect interpretability of deep learning for EEG decoding. Our findings emphasize that employing a network architecture that aligns with the nature of the data can lead to more interpretable features. Moreover, our results suggest that visualizing early network layers results in features that are closer to the data, while late layers to more abstract ones. These insights encourage the use of deep learning for EEG-based decoding while emphasizing the importance of choosing an appropriate network architecture and feature extraction methods.

## Supplementary Material

Supplementary Material

## Data Availability

The data and code for this study can be made available upon request.
