# Supplementary material for: What does my network learn? Assessing interpretability of deep learning for EEG
Source: Imaging Neurosci (Camb). 2025 Dec 2;3:IMAG.a.1033. doi: 10.1162/IMAG.a.1033 (PMC12673218; doi:10.1162/IMAG.a.1033)
Supplement: Supplementary Material [file IMAG.a.1033_supp.pdf]

Supplementary Methods

1. Deep Learning Important FeaTures (DeepLift)

DeepLIFT (Shrikumar et al., 2019) is a newer yet less commonly applied feature extraction method in EEG research. It assigns each neuron a contribution score by comparing its activation to activation for a reference input (set to zero in the current work) and backpropagating these scores through the network. The change in a target activation relative to its reference is decomposed as the sum of contributions from all input differences:

$$\Delta t = \sum_i C_{\Delta x_i \Delta t} \qquad (1)$$

where $\Delta x_i = x_i - x'_i$ is the difference between the activation neuron *i* at layer *x* (e.g. input layer) and activation to the reference input, $C_{\Delta x_i \Delta t}$ is the contribution of activation of *i*th neuron in layer x to the change in the target neuron $\Delta t = t - t'$.

Supplementary Figures

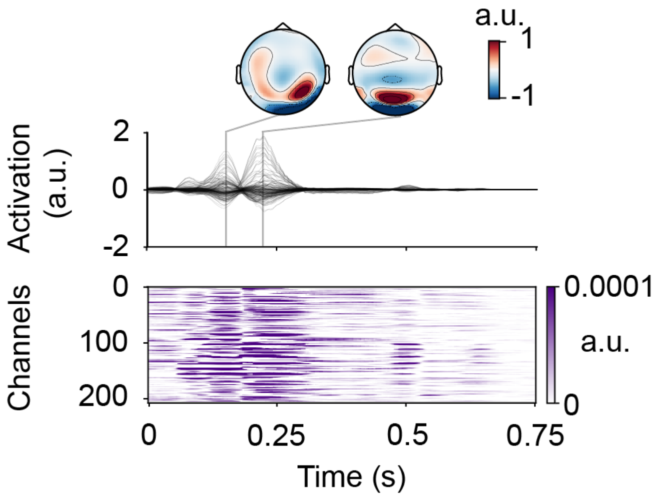

Supp. Fig. 1: Control analysis of feature visualization for the visual dataset using EEGNet with DeepLIFT: Darker colors corresponding to higher values indicate important channel-time point pairs for the network's prediction on the visual dataset. The values plotted as topographic maps have been transformed with surface Laplacian to emphasize spatial patterns.

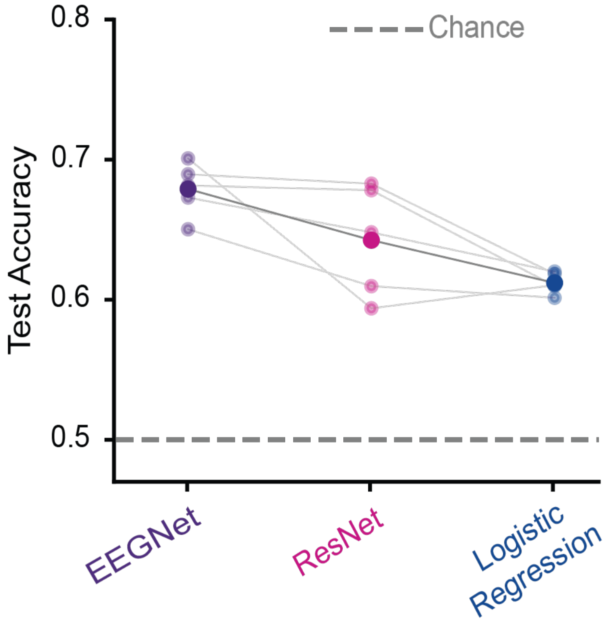

Supp. Fig. 2: Control analysis with an open-access dataset to assess model generalizability. We used the dataset from (Haupt, Garret, and Cichy, 2025), which includes N=43 participants and 1024 trials per condition per participant, for a total of N=88112 trials. Shown are the 2-class decoding results of EEGNet (purple), ResNet (pink), and logistic regression (blue) models. Each small (semi-transparent) dot represents the accuracy score obtained for test set data in one cross-validation fold, the big opaque dot represents the mean of all folds, and the gray dash line indicates chance level (0.5).

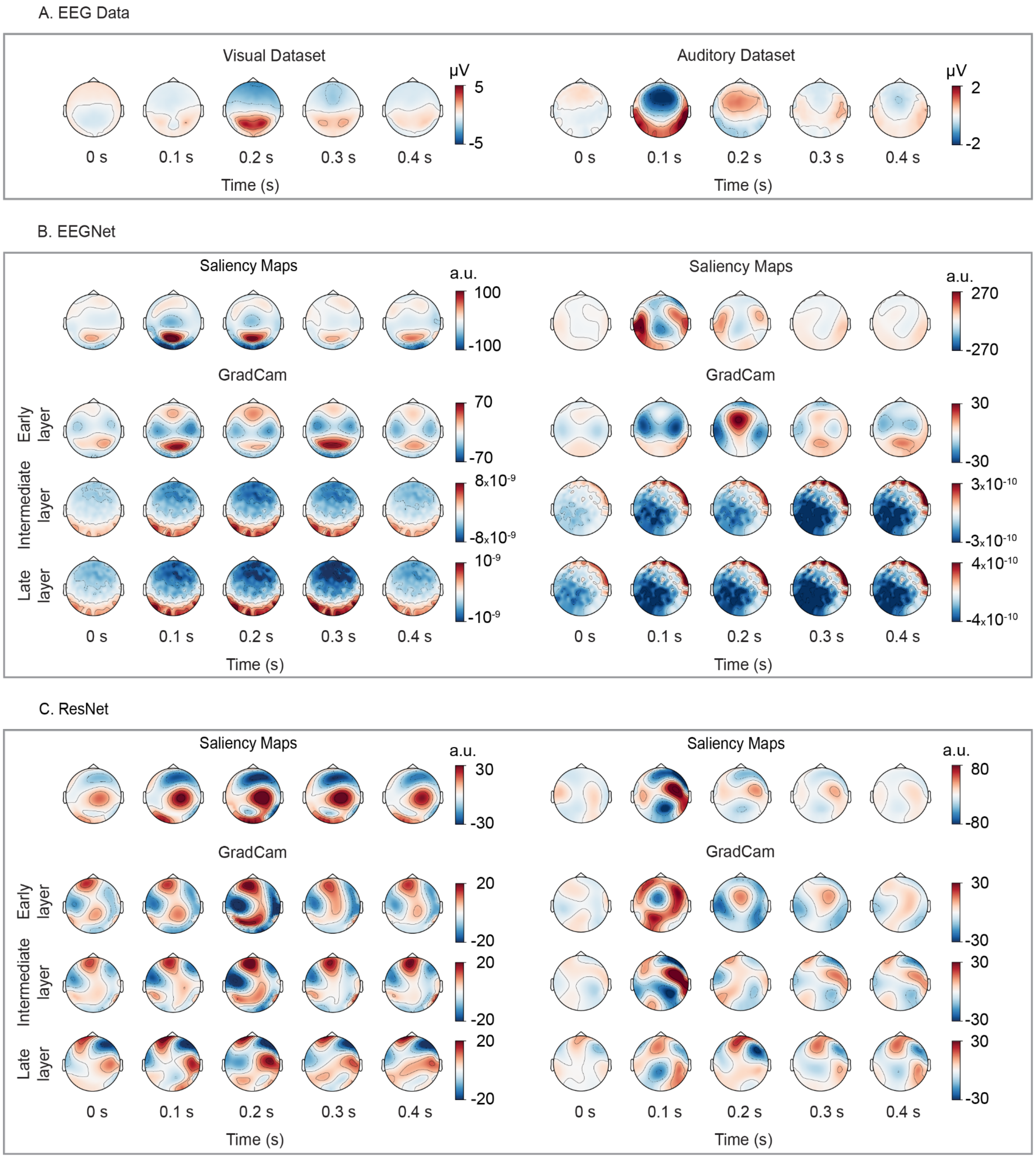

Supp. Fig. 3: Time course of EEG topographies and learned features. (A) EEG data for (left) the visual dataset and (right) the auditory dataset (post-stimulation interval). (B) EEGNet and (C) ResNet features extracted with (top) saliency method and (bottom)

GradCam method. In panels (B-C), the left side of each panel shows the features for the visual dataset, the right side shows features for the auditory dataset.

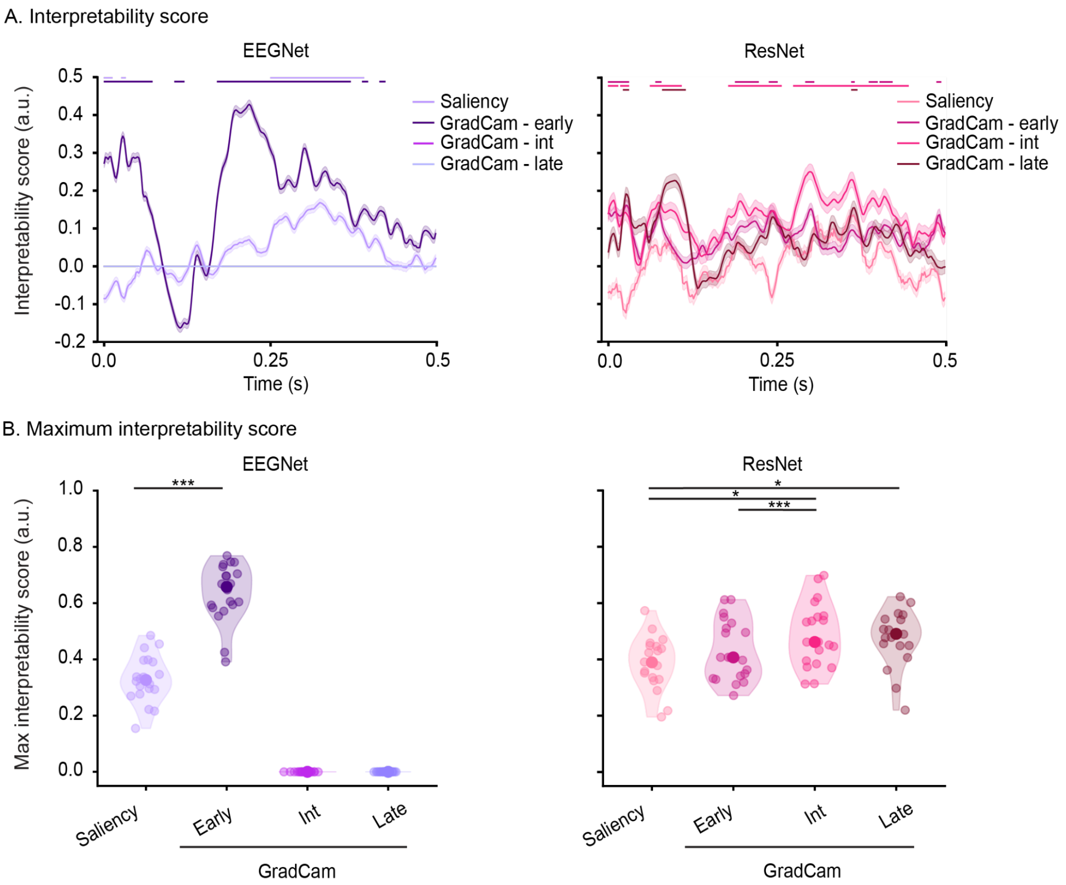

Supp. Fig. 4: Interpretability score for post-stimulation interval in the auditory dataset. (A) Time course of interpretability scores of features extracted by saliency and GradCam from early, intermediate (int), and late layers. Horizontal bars indicate the time points significantly different from zero after FDR correction ($p < 0.05$, non-parametric Wilcoxon signed-rank test). (B) Maximum interpretability scores per network and feature visualization. Scores of each feature extraction method and layer were statistically compared using the Wilcoxon paired-rank test. Bars on top of violin plots indicate which conditions were compared, and stars show statistically significant differences (* $p < 0.05$, ** $p < 0.01$, *** $p < 0.001$). In all panels, purple and pink colors represent EEGNet (left) and ResNet (right), respectively.

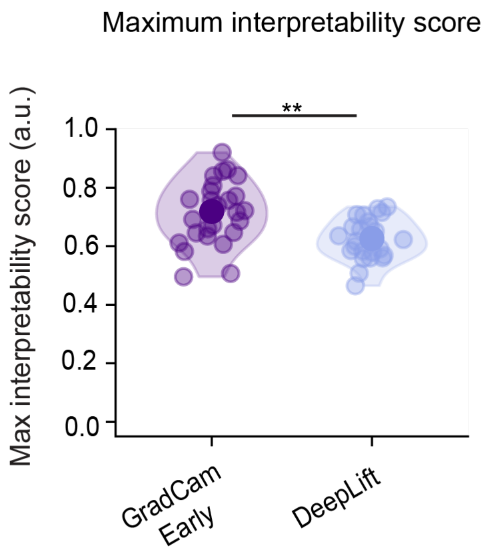

Supp. Fig. 5: Maximum interpretability scores of features extracted with GradCam from the early layer (left) and DeepLift (right) for the visual dataset. The interpretability score using DeepLIFT (Supp. Methods 2) was 0.62 ± 0.013 which was significantly lower than GradCam (0.72 ± 0.02) ($z = -3.14$, $p = 0.0017$). Each dot in panel B corresponds to one participant. Scores of each feature extraction method and layer were statistically compared using the Wilcoxon paired-rank test (* $p < 0.05$, ** $p < 0.01$, *** $p < 0.001$).

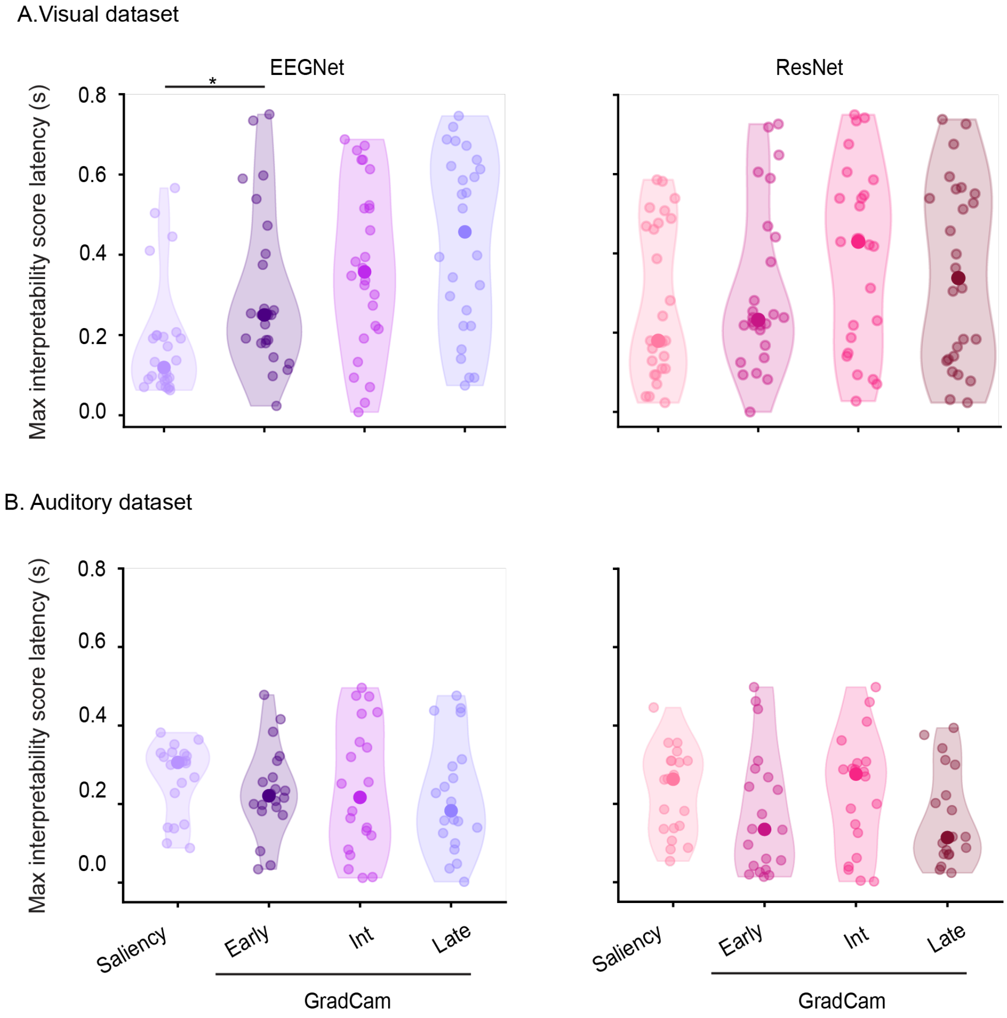

Supp. Fig. 6: Latency of maximum interpretability scores for (A) visual dataset and (B) auditory dataset. Latencies of each feature extraction method and layer were statistically compared with others using the Wilcoxon paired-rank test. Bars on top of violin plots indicate which conditions were compared, and stars show statistically significant differences (* $p < 0.05$). In all panels, purple and pink colors represent EEGNet (left) and ResNet (right), respectively. Each semi-transparent dot represents a participant, and the opaque dot corresponds to the group's median. We found that the latency of the maximum interpretability score was earlier in saliency maps than early layer GradCam

layers for EEGNet (left, $z = -2.3$, $p < 0.05$), while for ResNet, this comparison was not significant (right, $z = -0.8$, $p > 0.05$). See Table 2 for values, Table 3 for statistics.

Supplementary Tables

	Visual			Auditory		
	Train	Validation	Test	Train	Validation	Test
EEGNet	0.59 ± 0.02	0.50 ± 0.01	0.52 ± 0.04	0.75 ± 0.01	0.72 ± 0.04	0.69 ± 0.03
ResNet18	0.95 ± 0.04	0.5 ± 0.04	0.53 ± 0.04	0.83 ± 0.09	0.66 ± 0.03	0.63 ± 0.04
Logistic Regression	0.46 ± 0.02	0.48 ± 0.04	0.44 ± 0.07	0.63 ± 0.01	0.65 ± 0.03	0.59 ± 0.03

Supp. Table 1: Performance of EEGNet, ResNet18 and logistic regression for the respective task on the visual and auditory datasets. Reported values are mean ± standard error across participants.

	EEGNet				ResNet			
	Saliency	GradCam Early	GradCam Int	GradCam Late	Saliency	GradCam Early	GradCam Int	GradCam Late
Visual Dataset	0.18±0.03	0.3±0.04	0.37±0.04	0.43±0.04	0.27±0.04	0.3±0.04	0.4±0.04	0.35±0.06
Auditory Dataset	0.27±0.02	0.23±0.03	0.24±0.04	0.22±0.03	0.23±0.03	0.18±0.04	0.23±0.03	0.16±0.03

Supp. Table 2: The latencies of maximum interpretability scores across CNNs and feature extraction methods and layers for the (top row) visual and (bottom row) auditory datasets. Reported values are mean ± standard error in seconds across participants.
